# Influence of Size on the Microstructure and Mechanical Properties of an AISI 304L Stainless Steel—A Comparison between Bulk and Fibers

**DOI:** 10.3390/ma8020451

**Published:** 2015-01-29

**Authors:** Francisco J. Baldenebro-Lopez, Cynthia D. Gomez-Esparza, Ramon Corral-Higuera, Susana P. Arredondo-Rea, Manuel J. Pellegrini-Cervantes, Jose E. Ledezma-Sillas, Roberto Martinez-Sanchez, Jose M. Herrera-Ramirez

**Affiliations:** 1 Centro de Investigación en Materiales Avanzados (CIMAV), Laboratorio Nacional de Nanotecnología, Miguel de Cervantes 120, Chihuahua CP 31109, Mexico; E-Mails: francisco.baldenebro@uas.edu.mx (F.J.B.-L.); cynthia.gomez@cimav.edu.mx (C.D.G.-E.); jose.ledezma@cimav.edu.mx (J.E.L.-S.); roberto.martinez@cimav.edu.mx (R.M.-S.); 2 Facultad de Ingeniería Mochis, Universidad Autónoma de Sinaloa, Prol. Ángel Flores y Fuente de Poseidón, S.N., Los Mochis, Sinaloa CP 81223, Mexico; E-Mails: rmn1779@gmail.com (R.C.-H.); susypao79@gmail.com (S.P.A.-R.); manuel.pellegrini@ingenieria.lm.uasnet.mx (M.J.P.-C.)

**Keywords:** AISI 304L stainless steel, fibers, mechanical properties, microstructure

## Abstract

In this work, the mechanical properties and microstructural features of an AISI 304L stainless steel in two presentations, bulk and fibers, were systematically studied in order to establish the relationship among microstructure, mechanical properties, manufacturing process and effect on sample size. The microstructure was analyzed by XRD, SEM and TEM techniques. The strength, Young’s modulus and elongation of the samples were determined by tensile tests, while the hardness was measured by Vickers microhardness and nanoindentation tests. The materials have been observed to possess different mechanical and microstructural properties, which are compared and discussed.

## 1. Introduction

Austenitic stainless steels, particularly AISI 304L, usually have excellent corrosion resistance, good weldability and formability, good resistance to hydrogen embrittlement, in addition to high ductility and toughness. However, they have relatively low yield strength in the annealed state [1]. There are various strengthening mechanisms for austenitic stainless steels, such as grain refining, transformation strengthening and work hardening, converting them in materials widely used in engineering applications, such as in the manufacturing, nuclear, chemical, oil and petrochemical, and food industries, as well as the medical industry for biomedical implants [1,2]. Within the 300-series austenitic stainless steels, the 304 grade is the most commonly used due to its superior low temperature toughness, as well as its corrosion resistance [3]. Recently there has been an enormous amount of research addressing the improvement of the mechanical properties of austenitic stainless steel [4,5,6,7,8] without lowering corrosion resistance [9,10,11,12]. Many experimental studies have focused on AISI 304 and AISI 304L at elevated temperatures [13,14,15], under thermo-mechanical and cycle fatigue conditions [16,17,18,19,20,21,22], under creep conditions [23] and ductility loss of hydrogen-charged steel [24].

Bulk stainless steels are commonly produced by hot-rolling, followed by cold-swaging and annealing processes. One great advantage of fibers and metallic wires is that they show very high strength values and consistent properties, more so than any ceramic fibers. Conventional wire drawing methods are quite reasonable for producing steel wires with diameters all the way down to 100 μm, while diameters reaching to 10 μm or less can be obtained by the so-called Taylor process [25]. Steel fiber combines flexibility of a traditional textile fiber with high temperature resistance of steel. Stainless steel fibers are also resistant to mechanical stress, in particular to shearing stress, as opposed to glass, ceramic or carbon fibers, and they are resistant to corrosion. Stainless steel fibers have an excellent resistance to high temperatures.

The application fields of stainless steel fibers are the aircraft industry (embedded in an aluminum matrix), aerospace industry (along with boron, borsic and molybdenum fibers embedded in aluminum and titanium matrix), and rocket engines (within nickel alloys matrices) [25]. Steel wire is a common commercial reinforcement material, more for concrete than for metals or polymers. Steel fibers are commonly used as reinforcement in tires.

Fracture of metallic filaments differs in many respects from fracture of bulk samples. Particular manufacturing processes, such as drawing, melt spinning or crystallization from the vapor phase for fibers, that are needed to obtain their small lateral dimensions, may introduce specific defects and textures, and have influence on fracture behavior [26].

So far, a few research efforts have been performed on the comparison of the mechanical and microstructural properties of stainless steel bulk and fibers. Measurement of mechanical properties is a major part of the domain of materials characterization, therefore, in the present work, the mechanical properties and microstructural features of an AISI 304L stainless steel in two presentations, bulk and fibers, were systematically studied in order to establish the relationship between the microstructure and the mechanical properties.

It is noteworthy that the 304L stainless steel fibers were tested individually in a Universal Fiber Tester, capable of conducting tensile, relaxation, creep and fatigue tests on very small diameter filaments, with a resolution of 0.01 g.

## 2. Experimental Section

The materials for this study were commercial AISI 304L stainless steel samples, both in bulk and fiber states. The chemical composition of the researched materials was performed by inductively coupled plasma atomic emission spectroscopy using an iCAP 6500 Thermo Electron spectrometer (Chihuahua, México), as well as an EA 1110 CHNS-O Elemental Analyzer (Chihuahua, México) from CE Instruments. The microstructural features and fracture of both kinds of materials were characterized by scanning electron microscopy (SEM, Chihuahua, México), using a JEOL JSM7401F microscope (Chihuahua, México). Phase and precipitate composition were identified by transmission electron microscopy (TEM, Chihuahua, México) using a JEOL-JEM2200FS microscope (Chihuahua, México). X-ray diffraction (XRD, Chihuahua, México) measurements were performed on the specimens for phase identification, using a Panalytical X’Pert PRO diffractometer with Cu Kα radiation (λ = 0.15406 nm). The XRD patterns were indexed with X’Pert HighScore Plus software containing PDF-2 files database. Single fibers were subjected to tensile tests at room temperature using a universal fiber tester developed originally by Bunsell *et al.* [27], equipped with a load cell of 250 g calibrated from 0 to 100 g, with a 0.01 g of precision. The fiber specimens were glued to card supports so as to give a gauge length of 30 mm. The card protected the fibers from the machine grips. The tests were conducted at a strain rate of 4.0 × 10^−3^·s^−1^. Data acquisition used a PC linked to the fiber tester via a National Instrument interface card and WinATS 6.2 software from Sysma. In order to normalize the stress, the diameter of each fiber was systematically measured before each test by a Mitutoyo LSM-500S laser device (Chihuahua, México), with an accuracy of 0.01 µm. The calibration of this device was performed using some fibers whose diameter was measured by SEM. Tension tests on bulk specimens were carried out at room temperature, according to the ASTM-E8M standard [28]; an Instron 4469 universal testing machine with a load cell of 50 kN was used. A strain rate of 4.0 × 10^−3^·s^−1^ and a specimen gauge length of 30 mm were used. Yield strength, ultimate tensile strength and fracture strain of the specimens were calculated from the stress-strain curves obtained; an extensometer and a linear regression were used for obtaining the Young’s modulus. For porosity measurements, an optical microscope (OM, Chihuahua, México) Olympus PMG3 and an Image-Pro Plus image analyzer (Chihuahua, México) were used. Fracture analysis for fibers was carried out through SEM observations, while for bulk materials it was carried out through OM. Hardness of both specimens was measured using the Vickers microhardness method on the longitudinal and cross sections of polished samples. Nanoindentation tests were carried out by an Agilent Nano Indenter G200 (Chihuahua, México), using the G-series XP cycles interactive indentation mode, using a diamond Berkovich indenter tip with a radius of 20 nm, strain rate target of 0.05 s^−1^, harmonic displacement target of 1 nm and a frequency target of 75 Hz. The fibers were vertically and horizontally embedded in an epoxy resin and cured in a plastic mold; after curing, the fibers were hand polished in order to provide a smooth exposed surface and measurements were performed in their cross and longitudinal sections.

## 3. Results and Discussion

### 3.1. Chemical Composition

The chemical composition of the stainless steel samples (bulk and fibers) is given in Table 1. It can be seen that the composition conforms to the AISI 304L standard.

**Table 1:** Chemical composition of the bulk and fiber specimens of 304L austenitic stainless steel (wt.%).

Sample	C	Si	Mn	Cr	Ni	P	S	Fe
Bulk	0.023	0.278	1.488	18.163	8.214	0.026	0.024	Balance
Fiber	0.029	0.389	1.406	19.756	11.216	0.013	0.005	Balance
ASTM A240 [29]	0.030	0.750	2.000	18.000–20.000	8.000–12.000	0.045	0.030	Balance

### 3.2. Microstructural and Structural Characterization

**Figure 1:**
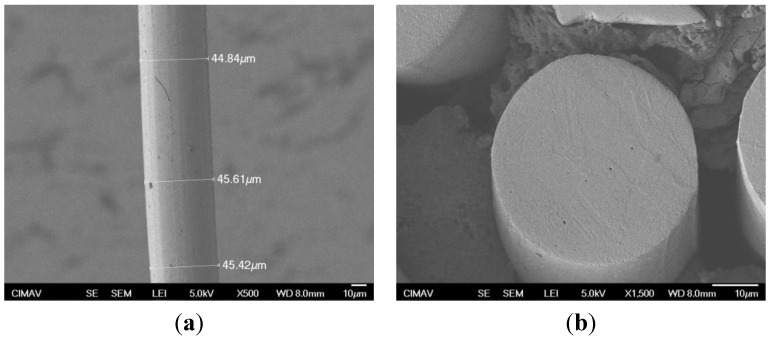
SE-SEM micrograph of individual 304L stainless steel fibers: (**a**) longitudinal and (**b**) cross-section.

Figure 1 shows SEM micrographs of an as-received 304L stainless steel fiber. Fibers appear smooth and are approximately circular; a circular cross-section was considered for stress calculation. They do not apparently present surface defects, except some longitudinal striations along their axis, produced by the manufacturing process. An average diameter of 45.29 ± 0.30 μm was determined, as evidenced in Figure 1a.

**Figure 2:**
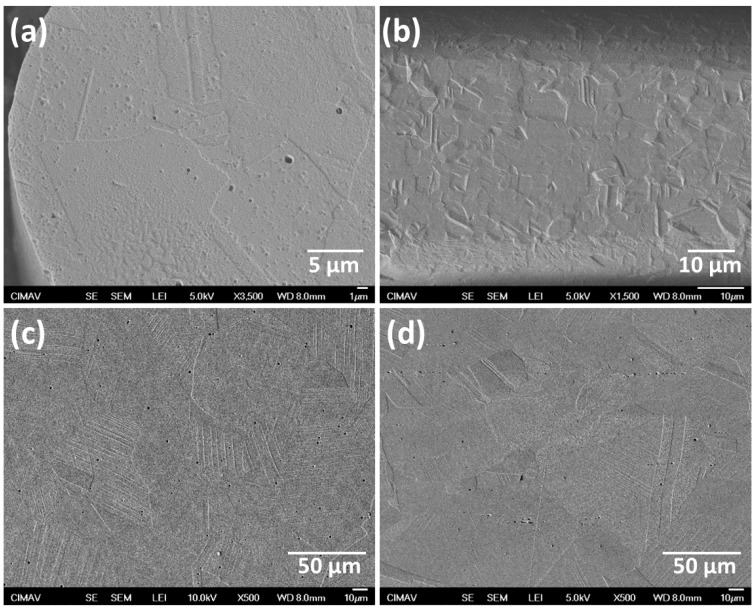
SE-SEM images of the AISI 304L samples: (**a**) cross; and (**b**) longitudinal sections of fibers; and (**c**) cross; and (**d**) longitudinal sections of bulk material.

The SEM observations revealed that both fibers (Figure 2a) and bulk (Figure 2d) specimens possess the same polygonal microstructure, similar to the cold-worked AISI 304L condition, with irregular austenite grains and dispersed carbide particles. The cold-work in austenitic stainless steels induces the martensitic transformation, and different martensite morphology can be formed. In Figure 2, slip bands in the austenite matrix are observed, which is indicative of the presence of martensite generated during the cold-work process. Due to the micrometer size of fibers, the micrographs are presented at different magnifications in comparison with those of the bulk material. It is important to notice that there exists a significant difference in grain size between both materials; a reduction of diameter during the fiber processing leads to the refinement of grain size.

**Figure 3:**
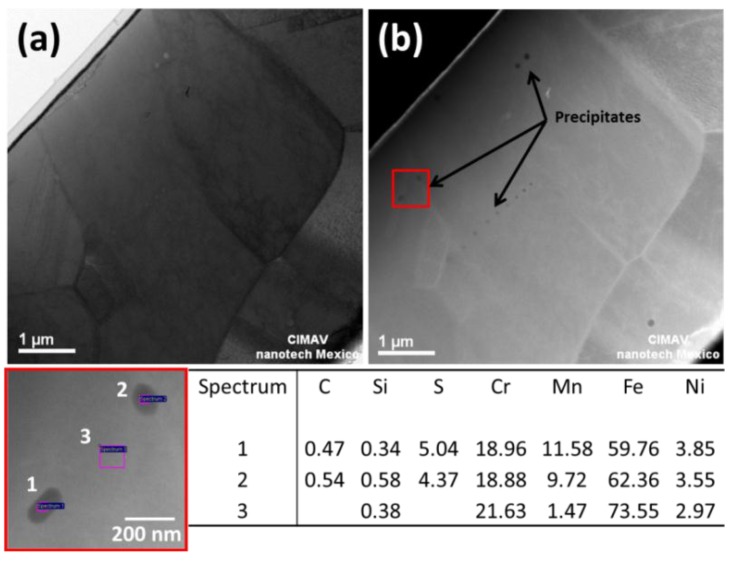
TEM micrographs: (**a**) bright field; and (**b**) Z-contrast images of a fiber sample showing the presence of nanoscale precipitates.

There are several strengthening mechanisms for austenitic stainless steels, such as grain refining and work hardening. Transmission electron microscopy (TEM) was used to examine the microstructural features of steel fibers. Thin foils from the specimens were prepared by focus ion beam (FIB) methods. The examination revealed a micrometer and sub-micrometer microstructure (Figure 3a). In addition, the presence of nanosized precipitates was found (Figure 3b). According to the EDS-TEM results presented at the bottom of Figure 3, the formation of nanosized carbides during the fiber processing is suggested. The presence of nanocrystalline phases in the fibers can influence their mechanical properties.

**Figure 4:**
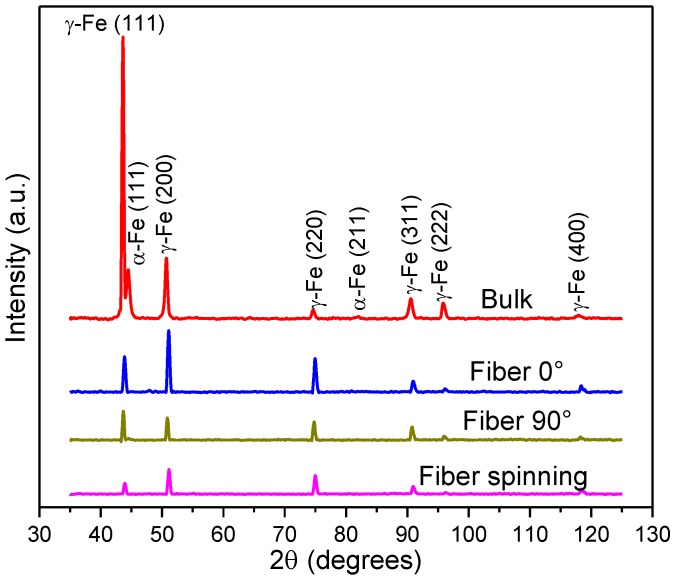
XRD patterns of the bulk and fiber samples.

XRD patterns of the studied samples are plotted in Figure 4 for the 2θ region from 35 to 125 degrees, with the Bragg reflections indexed. They revealed that both bulk and fiber samples have the same crystalline structure. During plastic deformation of austenitic stainless steels at room temperature, the martensitic transformation occurs from the austenite phase. The structure observed in the diffraction peaks consists of a mixture of bcc and fcc phases, corresponding to the characteristic AISI 304L α-Fe (martensite) and γ-Fe (austenite) phases, respectively. Nevertheless, the XRD patterns of AISI 304L samples in different states present a remarkable difference in intensity. The peaks of the bulk sample are sharp, while the peaks of the fiber pattern exhibit a significant shortening that indicates a crystal size refinement. The diameter size reduction of fibers produced by cold-working causes lower intensity of austenitic peaks (γ-Fe) in comparison to the bulk sample, while diffraction peaks of α-Fe phase cannot be easily found due to the fine crystal size. The crystal size and microstrains were determined by standard Scherrer equation. The found values of crystal size were 51.5 µm and 2.32 µm for the bulk and fiber samples, respectively. The microstrains of fibers (0.232%) exceeds the value of the bulk sample (0.181%). The calculated values of the lattice parameters for the bulk sample are a_α-Fe_ = 0.287 nm and a_γ-Fe_ = 0.359 nm, while for the fiber sample only the peaks corresponding to the γ-Fe phase were observed, with a lattice parameter of a_γ-Fe_ = 0.358 nm. A significant lattice mismatch was not found in both samples. On the other hand, the variation in the relative intensities of the main diffraction peaks of the bulk sample in comparison to the fiber 0°, fiber 90° and fiber spinning patterns, suggests the presence of crystallographic texture due to the processing route of the fibers.

### 3.3. Tensile Tests

**Figure 5:**
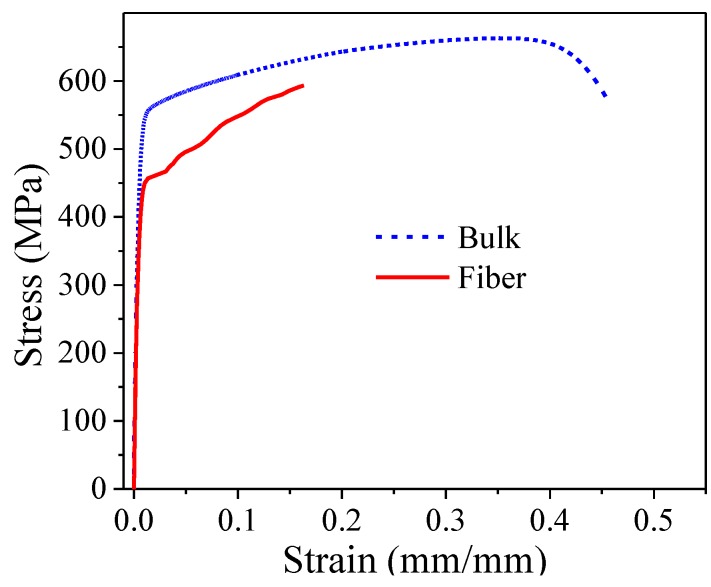
Stress-strain curves of the bulk and fiber samples.

The engineering stress-strain curves of both the bulk and fiber specimens are shown in Figure 5. It can be seen that the fiber samples show lower tensile mechanical properties than those of the bulk samples; the results are summarized in Table 2. The parabolic shape of the bulk specimen curve indicates that strain hardening occurs throughout the duration of the stress application, but such an amount of strain hardening for a given increment of stress decreases as stress increases. Concerning the fibers, instead of a parabolic-shaped curve, the stress increased monotonically up to failure, which means that there is always a strain hardening of the fiber structure. It is clear that the bulk mechanical properties exceed those of the fibers.

An improvement of yield stress (σ_y_) and Young’s modulus (E) in the fibers was expected due to grain refinement in comparison to the bulk sample, however the result was opposite to that expected. During cold-work crystalline defects like dislocations and porosity increase with the degree of deformation and decrease the mechanical properties. In addition, the sample sizes (diameter of 45.29 μm for fibers and 6.41 mm for bulk) affect the results of mechanical tests. The coupled effect of crystalline defects and tested area size of samples is evident in the reduction of mechanical properties of fibers evaluated in macro-scale in comparison to the bulk sample.

**Table 2:** Mechanical properties of 304L stainless steel samples.

Sample	σ_y_ (MPa)	σ_max_ (MPa)	ε (%)	E (GPa)	HV	
					Long-section	Cross-section
Bulk	492 ± 4.5	663.07 ± 9.1	45.91 ± 3.7	175.44 ± 10.7	273.53 ± 5.1	256.53 ± 7.2
Fiber	389 ± 7.2	597.47 ± 10.7	16.33 ± 2.8	162.76 ± 8.9	151.73 ± 9.1	169.12 ± 2.0

**Figure 6:**
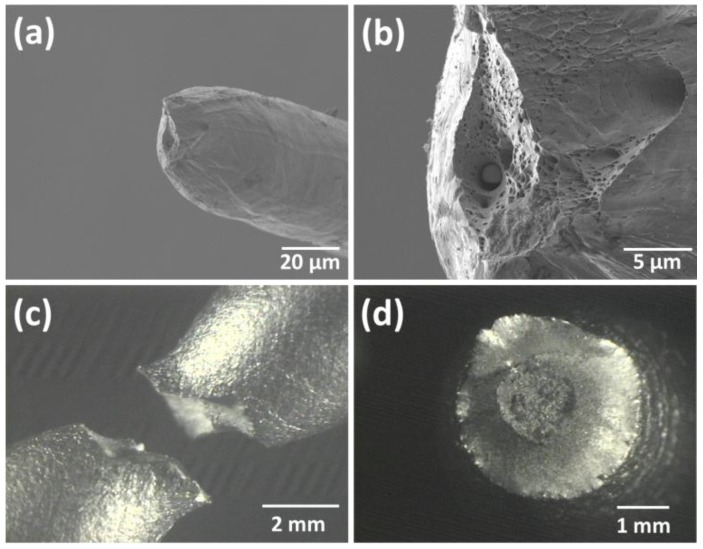
Fracture surface morphologies of tensile tested (**a**); and (**b**) fiber; and (**c**) and (**d**) bulk material.

The tensile fracture morphology of the samples was analyzed by optical microscopy (Figure 6). Necking and neck propagation were observed for both materials, which are associated with ductile materials (Figure 4a,c). Ductile fractures are characterized by tearing of metal along with appreciable gross plastic deformation. Ductile tensile fractures in most materials have a gray and fibrous appearance [12]. The quantity and size of pores after tensile tests can be seen in Figure 4b,d, respectively for fiber and bulk specimens; in the case of fibers, the fracture surface contains many honeycomb-like deep dimples, distributed homogeneously throughout the whole surface.

**Figure 7:**
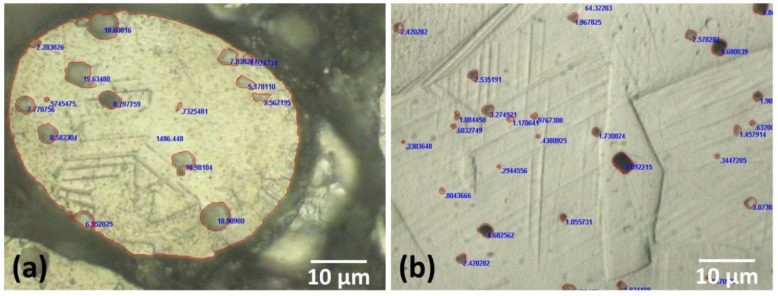
Optical microscopy images showing the measured diameter size of pores, represented by open red circles: (**a**) fiber; and (**b**) bulk specimens.

In order to determine the porosity percentage of the studied materials, optical microscopy measurements were conducted for the polished cross-section of samples (Figure 7). The bulk sample porosity was found to be 2.13%, while the fiber porosity was 5.84%. The mean pore diameter was 1.46 ± 0.21 µm and 4.21 ± 0.88 µm for bulk and fiber samples, respectively. Hence, it is evident that a greater volume fraction of porosity has a detrimental effect on the mechanical response of fibers, in comparison to the bulk condition. Porosity is known to have a significant effect on the mechanical properties of metals, in particular on the yield stress, ultimate tensile strength and fracture strain, as shown by the results in Table 2. In the case of Young’s modulus, this is a material intrinsic property; nevertheless, in the present work a decrease in Young’s modulus was found due to porosity, especially for fibers. A similar behavior was reported by J. Qiao, *et al.* [30] for 316L stainless steel fibers, where the Young’s modulus decreased with increasing sample porosity. Furthermore, N. Chawla and X. Deng [31] found that increasing density results in an increase in Young’s modulus for sintered steels.

### 3.4. Microhardness and Nanoindentation

Vickers microhardness tests were conducted in both kinds of samples; the results are presented in Table 2, which shows that the hardness of the bulk samples is higher than that of fibers. This lower microhardness of fibers can be due to their higher porosity; that is why nanoindentation measurements on cross-section were performed in order to determine hardness and Young’s modulus of bulk and fiber specimens. Regarding hardness measurements by nanoindentation, the bulk material presented a value of 4.19 GPa, while the fibers presented a value of 4.97 GPa; in both cases these results are significantly greater than those reported in Table 2: 2.52 GPa (256.53 HV) and 1.66 GPa (169.12 HV) for bulk and fibers, respectively. This can be explained by the fact that the nanoindentation measurements were carried out in flaw-free small regions, namely porosity-free, while Vickers microhardness measurements were performed over large regions that might contain porosity. In reference to the Young’s modulus, the values found by nanoindentation for the bulk and fiber specimens were 181.08 and 198.04 GPa, respectively, which are close to that reported for the AISI 304L stainless steel (193 GPa) [3]. According to Young’s modulus results obtained by tensile tests (Table 2) and nanoindentation, the values for bulk material are similar by both techniques, but in the case of fibers, the value is less for tensile tests (115.93 GPa); this difference can be again explained by the large number of flaws that samples contain throughout their longitudinal section, especially in the case of fibers. The hardness and the Young’s modulus measured by nanoindentation are higher because the measurements were performed over defect-free areas.

The higher values in fiber properties measured by nanoindentation yield evidence that deformation of fibers during cold-work induces greater material strengthening, making it stiffer by reducing their deformation ability without reaching a completely brittle material.

## 4. Conclusions

In this work tensile, microhardness and nanoindentation tests were done on a 304L stainless steel in the form of bulk and fibers, in order to evaluate and compare the influence of the microstructure on their mechanical properties. Both presentations showed a similar microstructure and tensile fracture morphology. Tensile and hardness tests showed that the bulk sample has higher values of yield stress, maximal stress, Young’s modulus than fibers, elongation and hardness, as a result of their porosity and pore size. It was observed that the macroscopic (tensile tests) and microscopic (microhardness tests) properties of the fibers are sensitive to these defects generated during the material manufacturing process.

Throughout nanoindentation testing a very small volume of material could be tested. The superior mechanical properties of bulk and fibers obtained by nanoindentation, in comparison with those obtained by tensile tests and microhardness, are due to the capability of nanoindentation of performing measurements on a small and porosity-free area. With this technique, higher values of hardness and Young’s modulus on fiber state were reached in comparison to bulk condition. These results were expected due to the greater plastic deformation induced in fibers during their processing. On the other hand, opposite results were obtained by microhardness and tensile tests, as greater pore density in fibers led to a reduction in the mechanical properties of the researched material.

Smaller crystal and grain size of fibers, in comparison to bulk samples, had a significant effect on their mechanical behavior measured by nanoindentation.
